# Designing Focused Chemical Libraries Enriched in Protein-Protein Interaction Inhibitors using Machine-Learning Methods

**DOI:** 10.1371/journal.pcbi.1000695

**Published:** 2010-03-05

**Authors:** Christelle Reynès, Hélène Host, Anne-Claude Camproux, Guillaume Laconde, Florence Leroux, Anne Mazars, Benoit Deprez, Robin Fahraeus, Bruno O. Villoutreix, Olivier Sperandio

**Affiliations:** 1 Inserm UMR-S 973/MTi, University Paris Diderot, Paris, France; 2 CDithem Platform/IGM, Paris, France; 3 Inserm UMR-S 761, Institut Pasteur de Lille, Lille, France; 4 Université Lille 2, Faculté des Sciences Pharmaceutiques et Biologiques, Lille, France; 5 UMR-S940, Hôpital St Louis, Paris, France; University of California San Diego, United States of America

## Abstract

Protein-protein interactions (PPIs) may represent one of the next major classes of therapeutic targets. So far, only a minute fraction of the estimated 650,000 PPIs that comprise the human interactome are known with a tiny number of complexes being drugged. Such intricate biological systems cannot be cost-efficiently tackled using conventional high-throughput screening methods. Rather, time has come for designing new strategies that will maximize the chance for hit identification through a rationalization of the PPI inhibitor chemical space and the design of PPI-focused compound libraries (global or target-specific). Here, we train machine-learning-based models, mainly decision trees, using a dataset of known PPI inhibitors and of regular drugs in order to determine a global physico-chemical profile for putative PPI inhibitors. This statistical analysis unravels two important molecular descriptors for PPI inhibitors characterizing specific molecular shapes and the presence of a privileged number of aromatic bonds. The best model has been transposed into a computer program, PPI-HitProfiler, that can output from any drug-like compound collection a focused chemical library enriched in putative PPI inhibitors. Our PPI inhibitor profiler is challenged on the experimental screening results of 11 different PPIs among which the p53/MDM2 interaction screened within our own CDithem platform, that in addition to the validation of our concept led to the identification of 4 novel p53/MDM2 inhibitors. Collectively, our tool shows a robust behavior on the 11 experimental datasets by correctly profiling 70% of the experimentally identified hits while removing 52% of the inactive compounds from the initial compound collections. We strongly believe that this new tool can be used as a global PPI inhibitor profiler prior to screening assays to reduce the size of the compound collections to be experimentally screened while keeping most of the true PPI inhibitors. PPI-HitProfiler is freely available on request from our CDithem platform website, www.CDithem.com.

## Author Summary

Protein-protein interactions (PPIs) are essential to life and various diseases states are associated with aberrant PPIs. Therefore significant efforts are dedicated to this new class of therapeutic targets. Even though it might not be possible to modulate the estimated 650,000 PPIs that regulate human life with drug-like compounds, a sizeable number of PPI should be druggable. Only 10-15% of the human genome is thought to be druggable with around 1000-3000 druggable protein targets. A hypothetical similar ratio for PPIs would bring the number of druggable PPIs to about 65,000, although no data can yet support such a hypothesis. PPI have been historically intricate to tackle with standard experimental and virtual screening techniques, possibly because of the shift in the chemical space between today's chemical libraries and PPI physico-chemical requirements. Therefore, one possible avenue to circumvent this conundrum is to design focused libraries enriched in putative PPI inhibitors. Here, we show how chemoinformatics can assist library design by learning physico-chemical rules from a data set of known PPI inhibitors and their comparison with regular drugs. Our study shows the importance of specific molecular shapes and a privileged number of aromatic bonds.

## Introduction

Protein-protein interactions regulate most aspects of Life and mapping these networks is nowadays one of the most difficult challenges in molecular medicine and biology. Aberrant PPIs contribute to most disease states and therefore represents a highly populated class of essentially untouched targets for drug discovery. While all PPIs may not be modulated by small drug-like compounds, among the about 650,000 interactions that regulate human life [1], a sizable number should be druggable [2]–[7], as suggested by the growing number of PPI systems successfully targeted by drug-like compounds, and the recent progress of two PPI drugs to clinical testing in humans[8]. Although a vast array of high-throughput, fragment-based and in vitro/in silico screening technologies have been developed over the last 15 years [9], the time and cost to chart PPI networks using these approaches frighten any corporate decision board or government funding body. Identification of PPI modulators is definitively challenging [3], [5]–[6], [10]–[11] due to the plasticity of some interfaces but most importantly to the unbalance between today's screening libraries and PPI inhibitors' chemical spaces [4], [12]–[18]. Hence, a possible avenue to minimize the biomolecular or in silico screening burden that is required to successfully target PPIs, is to design focused libraries enriched in PPI inhibitors to realign the chemical space window of compound collections with the chemical requirements of PPI inhibitors. This approach should not only reduce wastes by eliminating a priori compounds that are unlikely to impede/modulate protein-protein complex formations but also lead to enhanced potency or specificity of the binders. The focused library concept [19] used on regular targets (e.g enzymes, GPCRs) has however to be tailored to the singularity of PPIs. We advocate that a possible solution to this conundrum is to mine relevant drug-like PPI inhibitors and define a dedicated profile through the use of appropriate chemoinformatics and machine learning tools. Indeed, previous reports [3], [20]–[23] have highlighted certain “universal” physico-chemical features of PPI inhibitors, i.e., our present understanding is that the molecules tend to be larger than regular catalytic site inhibitors, they tend to be relatively hydrophobic and rigid while often containing aromatic groups [3], [21]–[24], suggesting that it should be possible to apply machine-learning and chemoinformatics methodologies on these molecules together with key molecular descriptors to design a PPI inhibitor profile and some PPI-inhibitor focused libraries [25]–[27]. Nevertheless, there is still some debate about whether such profile could or should be global (i.e PPI-independent) or target-specific (like for GPCRs or kinases). While it is clear that a global filter can not reduce the size of the initial collection as much as a target-specific filter, it has important advantages in the early discovery stages on this difficult target class (i.e., for many PPIs there are neither known small molecule inhibitors nor 3D structures to focus the collection in a conventional target-specific manner).

In the present study, we selected appropriate Dragon's molecular descriptors[28] on a learning data set composed of true PPI inhibitors and non-PPI inhibitors. We then ventured to build machine learning-based computer models able to predict a global and target-independent PPI inhibitor profile and transposed it into a computer program, PPI-HitProfiler. We applied our tool to focus several commercial compound collections to probe the concept and assess the level of size reduction of those databases. Most importantly, our program was further challenged on the experimental screening results of 10 PPIs downloaded from the PubChem Bioassay server. In addition, we carried out the in vitro screening of two chemical library subsets on the p53/MDM2 interaction within our CDithem drug discovery platform. Collectively, these experimental results confirm the robustness of our tool, which managed to discard more than half of the non-PPI inhibitors while identifying 70% of the true PPI inhibitors on those systems.

## Results/Discussion

### Construction of a machine-learning model to profile PPI inhibitors

**Figure 1:**
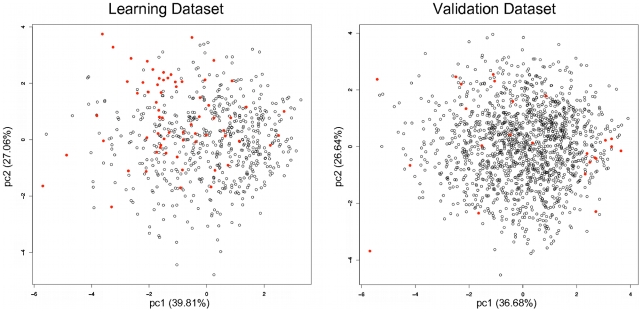
Principal Component Analysis (PCA) on the learning and validation data sets. The calculations were run using 7 physico-chemical, molecular weight, octanol/water partition coefficient, topological polar surface area, number of Hydrogen bond donors and acceptors, the number of rotatable bonds and the number of rigid bonds. PPI inhibitors are represented as red disks, and non-PPI inhibitors are represented as black circles.

We used a chemical fingerprint-based clustering approach to construct a chemically diversified learning data set ultimately composed of 66 validated drug-like PPI inhibitors (Figure S1) selected from the literature and of 557 non-PPI inhibitors obtained from the “small molecule” subset of the DrugBank [29]. This latter subset was chosen because historically it contains very few (if any) PPI inhibitors, and therefore represents a valuable pool of non-PPI inhibitors. Indeed, only 7 compounds on the whole DrugBank small subset (4857 compounds) had a Tanimoto index above 0.8 with one of the 66 PPI inhibitors. Further, to evaluate the level of physico-chemical overlap between PPI and non-PPI inhibitors, we ran a Principal Component Analysis (PCA) using key descriptors (referred to as physico-chemical PCA), namely molecular weight, octanol/water partition coefficient, topological polar surface area, number of Hydrogen-bond donors and acceptors, number of rotatable bonds and number of rigid bonds (Figure 1). The subspace spanned by the first two principal components (which account for more than 60% of the total variance of the global physico-chemical space) are comparable for PPI and non-PPI inhibitors on the learning data set (comparable range and variability). The coverage of the protein space corresponding to the 66 PPI inhibitors was also evaluated by considering the SCOP fold classes of the associated PPIs. The 66 PPI inhibitors span over 27 different PPIs and 21 different SCOP fold classes including various topological properties: mainly helix-based domain; mainly beta-strand domain; mix folding (helix + beta strand); and loop-binding groove domains (Figure S2).

On this learning data set, we initially computed the 1,666 Dragon molecular descriptors of E-Dragon (Dragon web version, http://www.vcclab.org/lab/edragon/), but eventually kept only the 357 most informative descriptors, were tested to construct several machine-learning methods, such as, Decision Trees (DT) and Support Vector Machines (SVM). The parameters of the learning models were optimized using a cross validation protocol such as to provide the best balance between enrichment (EF), sensitivity (Se) and specificity (Sp) on the learning data set. We then assessed these models on an independent validation data set (Figure S3), composed of 26 other PPI inhibitors (that were not present in the learning data set of 66 PPI inhibitors) and 2,000 decoys taken from the ChemBridge diversity set (www.chembridge.com). The 26 PPI inhibitors span over 5 different PPI and 5 different SCOP fold classes (Figure S4). Similarly to the analysis performed on the learning data set, the physico-chemical PCA ran on the PPI- and non-PPI inhibitors showed a fair overlap of the physico-chemical subspaces associated to the two first principal components (Figure 1).

From a methodological standpoint, we first observed that the selected machine learning techniques could be successfully applied to define/confirm a PPI inhibitor profile on the learning data set (Table 1). Clearly, all SVM kernels were very efficient at predicting the PPI inhibitor profile on the learning data set, with a sensitivity of 92% and a specificity of 100% in the case of the optimized sigmoid kernel. The two best DTs (1 and 2) also performed well on the learning data set with sensitivities of 85 and 76% and specificities of 70 and 77%, respectively.

**Table 1:** Prediction results of the five best machine-learning models.

Parameters	Data set	Se (%)	Sp (%)	EF
D.T.1	Learning	85	70	2.38
D.T.1	Validation	81	66	2.53
D.T.2	Learning	76	77	2.61
D.T.2	Validation	70	80	3.39
SVM Gaussian Kernel	Learning	89	100	9.44
SVM Gaussian Kernel	10-FCV	39	97	5.71
SVM Gaussian Kernel	Validation	33	85	2.23
SVM Sigmoid Kernel	Learning	92	100	9.29
SVM Sigmoid Kernel	10-FCV	42	93	3.83
SVM Sigmoid Kernel	Validation	33	81	1.77
SVM Polynomial Kernel	Learning	89	100	9.44
SVM Polynomial Kernel	10-FCV	33	98	5.77
SVM Polynomial Kernel	Validation	27	84	1.67

Representation of the different optimized machine learning models, two decision trees, and three SVM models. Se (sensitivity), Sp (specificity) and EF (Enrichment) values are given for both, the learning data set (66 PPI inhibitors +557 non-PPI inhibitors) and the validation data set (26 PPI inhibitors +2,000 non-PPI inhibitors). (10-FCV = 10-Fold Cross Validation).

**Figure 2:**
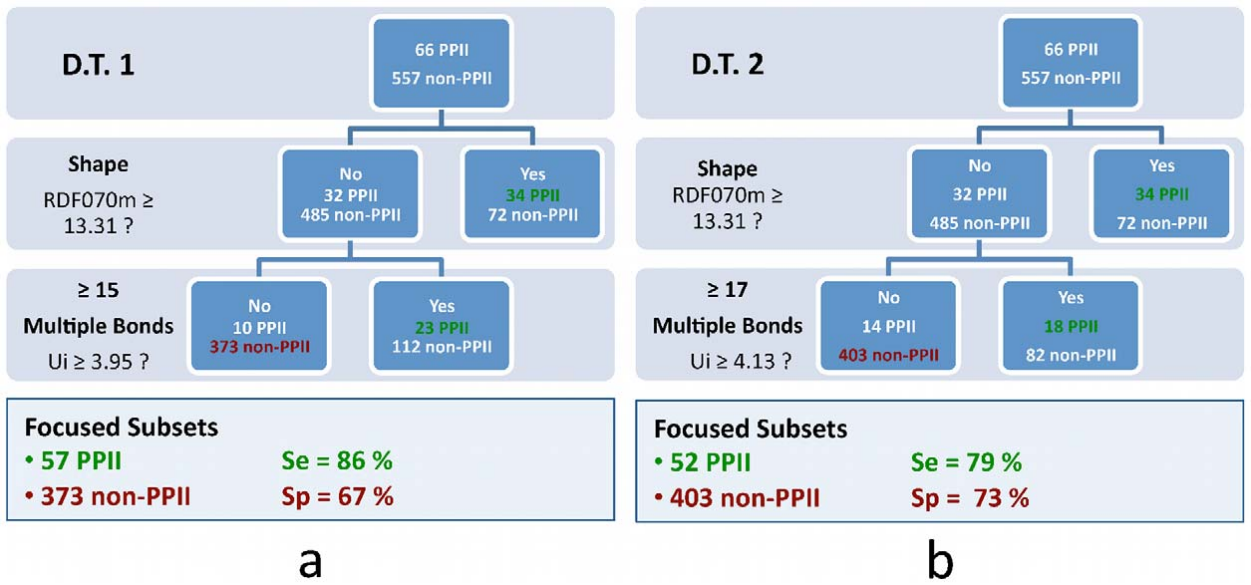
Representation of the two decision trees D.T.1 (panel a) and D.T.2 (panel b) on the learning data set (66 PPI inhibitors + 557 non-PPI inhibitors). The two decision trees share the same two descriptors RDF070m and UI. The two thresholds for RDF070m are identical (≥13.31) for D.T.1 and D.T.2 while the UI thresholds are different, ≥3.95 and ≥4.13 respectively for D.T.1 and D.T.2. The values for the corresponding sensitivity (Se) and specificity (Sp) are indicated for each DT.

**Figure 3:**
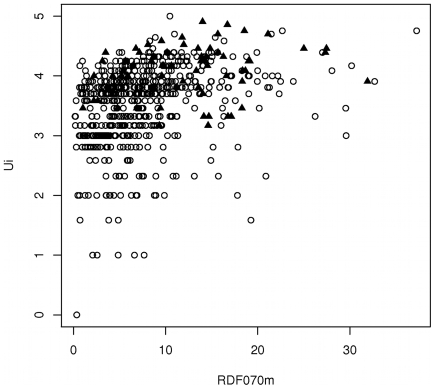
Correlation Plot between RDF070m and UI descriptors. Plot calculated on the 623 molecules of the learning data set: 66 PPI-inhibitors (dark filled triangles) +557 non-PPI inhibitors (dark circles). The two thresholds of the decision tree N°1 are RDF070m>13.31 and UI>3.95. This plot first highlights the poor correlation between RDF070m and UI (r^2^ = 0.34). Secondly, this shows that most of the PPI-inhibitors are either above the RDF070m threshold (13.31) or above the UI threshold (3.95).

Nonetheless, we have mostly considered the performances of the models on the validation data set (26 PPI inhibitors +2000 decoys), which conceptually represents a real-life assessment of the models. As seen on Table 1, the quality of the SVM-model predictions could not be maintained neither during the 10-fold cross validation (10-FCV) on the learning data set nor with the validation data set as shown by the obtained sensitivity, specificity and enrichment values. This clearly demonstrates an over training of the SVM models on the learning data set, regardless of the kernel used, and despite the cross validation-based optimization of the SVM parameters. On the contrary, D.T.1 and D.T.2 display more robust performances on the validation data set, with D.T.1 showing a sensitivity of 81% and a specificity of 70%, and D.T. N°2 showing a lower Se (70%) but a higher Sp (80.1%). These results show that in this application decision trees outperform support vector machines in predicting a PPI inhibitor profile on our independent validation data set. This behavior also brings the net advantage to provide the medicinal chemists with a comprehensive description of the relevant physico-chemical features required in the design or selection of PPI inhibitors. Indeed, decision trees can offer a significant advantage over SVM models, which result from the combination and transformation of all the descriptors and usually lack interpretability. In the present case, two decision trees were constructed in order to propose two ways of balancing specificity and sensitivity. The two best DTs were constructed with the two same Dragon descriptors, RDF070m and UI, though with different thresholds for Ui (≥3.95 and ≥4.13) (Figure 2). We observed a poor correlation between RDF070m and Ui (r^2^_RDF070m:UI_ = 0.34), which confirms that they provide low redundancy and good complementarities in discriminating PPI- from non-PPI inhibitors (Figure 3). RDF070m is a Radial Distribution Function (RDF(*r*)) descriptor weighted by the atomic masses using a sphere radius *r* of 7 Å as the associated probability distribution function, and Ui is the unsaturation index, directly correlated to the number of multiple bonds (double, triple and aromatic bonds).

RDF(*r*) descriptors are known as shape descriptors. They represent a radial distribution function of an ensemble of N atoms and can be interpreted as the probability distribution to find an atom in a spherical volume radius *r*:
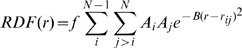
where *f* is a scaling factor, *N* is the total number of atoms, *A_i_* and *A_j_* are atomic properties associated with the atom *i* and *j* whose distance is defined by *r_ij_*. *B* is a smoothing factor. *f* and *B* were set to 0.007 and 100 Å^−2^ respectively. *A_i_* and *A_j_*, are in the case of RDF070m, the atomic weight of atom *i* and *j*, respectively. This family of descriptors is usually used as a multiple-value code calculated at different discrete distances (here we just use *r* = 7 Å) and can be weighted by various atomic properties, here the atomic weight, but it can be partial charges, polarizability, etc. These descriptors were successfully used to study active compounds on Vitamin D receptor [30], flavonoid compounds as inhibitors of aldose reductase [31] but more interestingly to predict 3D structures from their infra red spectra in which specific substructures are by definition associated to a specific signal, like the presence or absence of multiple bonds in a given region of the compounds [32]–[33].

**Figure 4:**
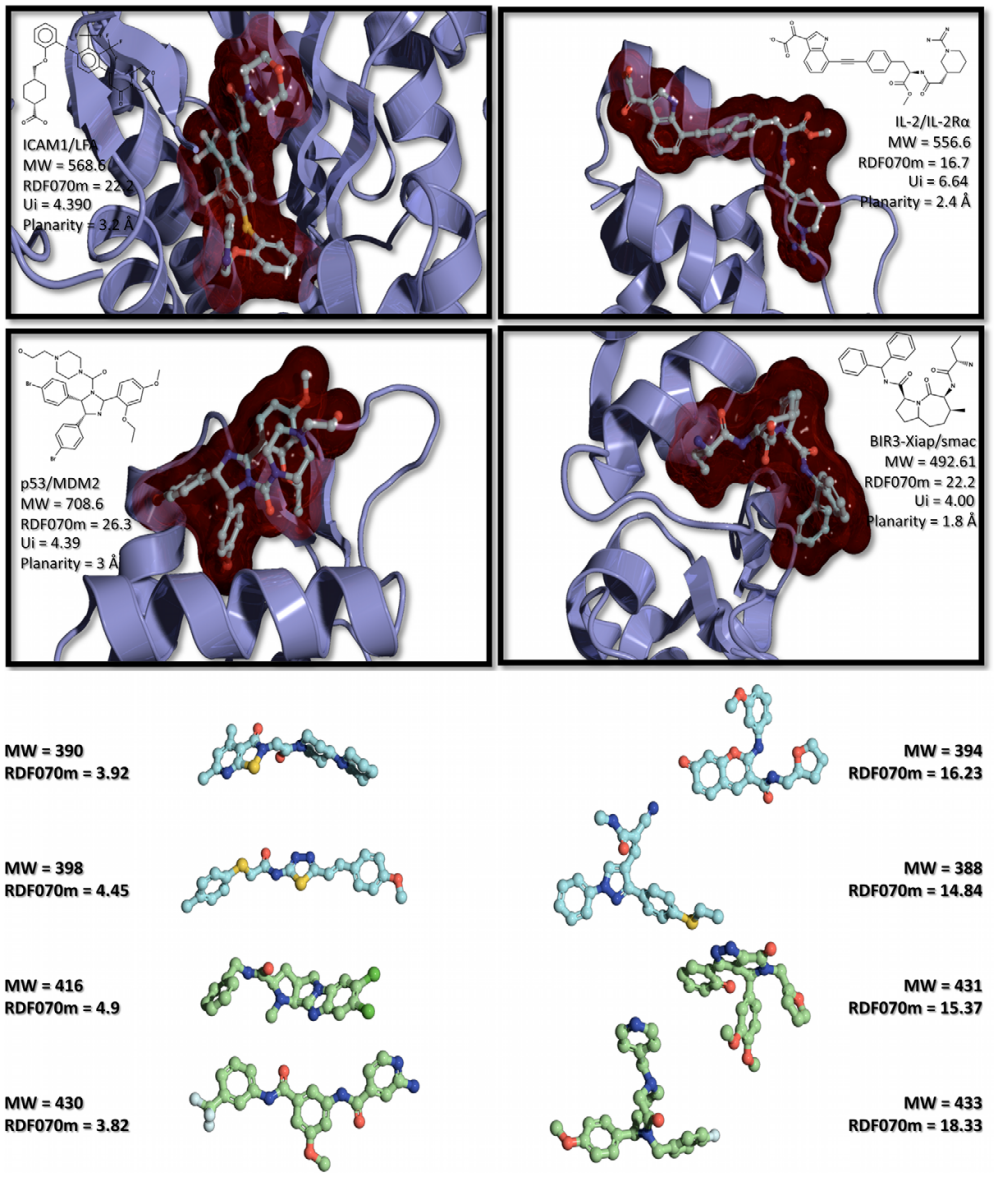
Effect of molecular shape on descriptor RDF070m. The RDF070m values have been calculated on 4 cocrystallized PPI inhibitors of the following PPIs: ICAM1/LFA, IL-2/IL-2Rα, p53/MDM2, and BIR3-Xiap/Smac. All 4 values are above the DTs threshold 13.31. The UI values are also indicated, as well as the planarity of the binding pocket (calculated from the PROTORP server, http://www.bioinformatics.sussex.ac.uk/protorp/). Also, on the panel below the calculated RDF070m values for 4 experimentally identified inhibitors (cyan) and 4 inactive compounds (green) of the CBFb/CBFa interaction taken from PubChemBioassay AID1434.

**Figure 5:**
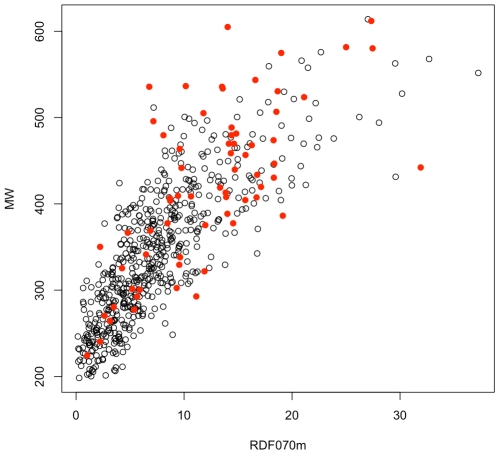
Correlation plot between RDF070m and MW on the learning data set (66 PPI inhibitors (Red disks) +557 non-PPI inhibitors (black circles)). This figure shows that correlations between RDF070m and MW are significant only for compounds below 400. At higher MW, RDF070m performs better than MW.

To illustrate the connection between the RDF070m descriptor and the molecular shape, we calculated the RDF070m values for 4 co-crystallized synthetic inhibitors taken from 4 different PPI complexes (1 protein of the PPI+1 synthetic inhibitor), namely ICAM1/LFA, IL-2/IL-2Rα, p53/MDM2, and Xiap-BIR3/smac complexes. We further calculated the values of RDF070m on 4 experimentally identified PPI inhibitors and 4 inactive compounds, all 8 taken from the screening of the PPI CBFb/CBFa interaction (PubChem Bioassay AID1434) (Figure 4). It is clear on Figure 4 that RDF070m tends to have higher values when the molecules have more ramifications and/or are star-, L-, or T-shaped. Conversely, I-shaped molecules have lower values. To further stress the prevalence of specific shapes observed within PPI inhibitors structures, we noticed that several of the p53/MDM2 inhibitors satisfying the “thumb-index-middle” finger-pharmacophore[34] that were present in our validation data set (26 PPI inhibitors) have also high values for RDF070m. It must be noted that even though RDF070m correlated partially with the molecular weight (MW), it is only true at lower MW (MW<400) (Figure 5). But, the combined descriptor obtained by dividing RDF070m by MW is still capable of significantly discriminating PPI inhibitors (p-value_RDF070m/MW_ = 5.74e-08). This is particularly important because RDF070m stands at the top of the DTs and therefore operates on the full data set. This demonstrates the information added by RDF070m to significantly separate the two populations (PPI inhibitors and non-PPI inhibitors) even at equivalent MW. Indeed, it can be seen on Figure 4 that even smaller compounds can have relatively high RDF070m values.

**Figure 6:**
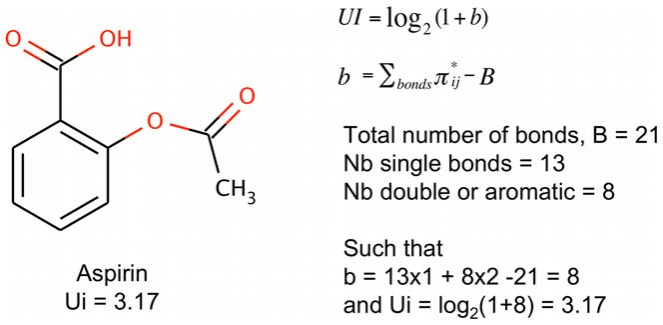
Calculation of UI on Aspirin. Ui_Aspirin_ = 3.17: aspirin has a total of 21 bonds, such that B = 21, 13 single bonds that have a contribution of 1 to b, and 8 double and aromatic bonds that have a contribution of 2 to b, such that the multiple bond count b is equal to b = 13x1+8x2 - 21 = 8. Thus Ui = log_2_(1+b) = log_2_(1+8) = 3.17.

The second yet unraveled descriptor, UI, depends exclusively on the number of multiple bonds:
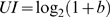
where, 
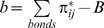
 is the multiple bond count, and *π*_ij_^*^ is the conventional bond order of the bond between atom *i* and atom *j* (*π*_ij_^*^ = 1 for single bonds, *π*_ij_^*^ = 2 for double and aromatic bonds, *π*_ij_^*^ = 3 for triple bonds), the summation being run over all B bonds. One can see that for single bonds, the *π*_ij_^*^ contribution cancels out with the term *B*, therefore making Ui relying exclusively on the *π*_ij_^*^ contribution of the multiple bonds. An example of the Ui calculation is given on Figure 6 with Aspirin. With such definition, one notices that the two above optimized thresholds associated with the two DTs (D.T.1_UI-threshold_≥3.95, D.T.2_UI-threshold_≥4.13), although being float values, can be traced back to a discrete number of privileged multiple bonds. Indeed, if one considers the number of triple bonds as negligible, which is the case with 0.1-0.6% of triple bonds on average on any given database, the two Ui thresholds correspond to a number of 15 and 17 multiple bonds (double or aromatic) respectively. This can be confirmed by the strong correlation observed between Ui and more explicit descriptors such as the number of multiple bonds (r^2^_UI:nBM_ = 0.95), the number of aromatic bonds (r^2^_UI:nAB_ = 0.92) and to a minor extent to the number of benzene-like ring (r^2^_UI:nBnz_ = 0.75), highlighting the importance of double and aromatic bonds. This is also coherent with previous observations[3],[6],[24],[34] about the more pronounced aromatic, hydrophobic and rigid character of PPI inhibitors.

By analyzing these results, we suggest that the two models (D.T.1 and D.T.2) we built bring complementary performances in terms of sensitivity and specificity. D.T.1 has a stronger ability to identify true PPI inhibitors (Se_D.T.1_ = 81%, Se_D.T.2_ = 70%) while D.T.2 has a higher level of discrimination towards non-PPI inhibitors (Sp_D.T.1_ = 66%, Sp_D.T.2_ = 80.1%). Therefore, the first tree would be more suited to operate on relatively small libraries (1,000 – 50,000 compounds) to maximize the chance of keeping a higher number of true PPI inhibitors, while the second tree will be useful to shrink large compound collections (over 50,000 compounds) with a higher efficacy, while keeping up to 70% of true PPI inhibitors.

Lastly, during the 20-fold cross validation (20-FCV) procedure used to choose the best descriptors involved in the DTs, other descriptors emerged, although much less frequenty than RDF070m and Ui. The first descriptor, ATS8m, could be used instead of RDF070m at the top of the tree (EF = 2.65, Se = 72.7%, Sp = 77.9%) in a very small minority of the 20-FCV configurations (2 times over 20). This descriptor is a Broto-Moreau's autocorrelation coefficient weighted by the molecular weight like RDF070m. It is based on a Dirac delta function center at an inter-atomic distance of 8 Å as opposed to RDF070m that is constructed in reference to an inter-atomic distance of 7 Å. Interestingly, RDF080m was found interesting for one case in the 20-FCV configurations (EF = 2.53, Se = 78.8%, Sp = 74.5%), highlighting also an inter-atomic distance of 8 Å. Concerning the second node of the tree, the descriptor PCR, could be used instead of Ui only on a minority of the 20-FCV configurations (2 over of 20)(EF = 2.33, Se = 77.3%, Sp = 72.0%), and correlated with Ui (r^2^ = 0.802). This descriptor is a walk and path count descriptor and more specifically the ratio of multiple path count over path count descriptor. This descriptor is, as Ui, linked to the ratio of multiple bonds with respect to the total number of bonds, but was found poorly efficient to discriminate true PPI inhibitors as compared to Ui.

Those results highlight the relevance and robustness of the chosen descriptors, RDF070m and Ui. Indeed, even when these descriptors were not retained as the very best ones (due to the unavoidably subsampling bias of the 20-FCV procedure), the alternative descriptors chosen brought a similar rather than orthogonal description.

### Implementation of the DTs into a computer program: PPI-HitProfiler

We then developed a computer program, named PPI-HitProfiler, to transpose our best DTs into a user-friendly command line package that takes as input any drug-like chemical library, calculates for each compound the two aforementioned descriptors, determines whether the compound satisfies the corresponding thresholds and generates a focused chemical library enriched in putative PPI inhibitors. As seen above, RDF070m and Ui are relatively simple descriptors to implement. This has been done using the Python-Pybel package[35] which is an object-oriented programming package allowing an easy manipulation of small compounds and of their main atomic properties.

### Assessment of PPI-HitProfiler on size reduction with commercial compound collections

To illustrate the benefit of using PPI-HitProfiler in terms of reducing the chemical collection size, we applied it on a drug-like version of the MayBridge Screening Collection (www.maybridge.com) filtered with our ADMET tool FAF-Drugs2[36]. From the 57,200 molecules initially present in this library, 31,107 molecules passed the soft ADMET filtering protocol. Subsequently, 17,162 molecules passed PPI-HitProfiler when using model D.T.1 and 13,799 for the model D.T.2 (Table 2). A similar evaluation carried out on the diversity set of the ChemBridge database that initially contained 50,000 compounds led to an intermediate library of 39,623 satisfying the ADMET filters and ultimately 12,866 compounds with PPI-HitProfiler-D.T.1 and 9,622 compounds with PPI-HitProfiler-D.T.2 (Table 2). In this latter case, the use of D.T.2 represents a size reduction of 76% from the ADMET version of the ChemBridge diversity set, and of 81% from the initial ChemBridge diversity set.

**Table 2:** Effect of PPI-HitProfiler on the size of two commercial collections.

MayBridge Screening Collection 57,200 Compounds		ChemBridge Diversity Set 50,000 Compounds	
ADMET filter 31,107 Compounds		ADMET filter 39,623 Compounds	
PPI-HitProfiler-D.T.117,162 Compounds	PPI-HitProfiler-D.T.213,799 Compounds	PPI-HitProfiler-D.T.112,866 Compounds	PPI-HitProfiler-D.T.29,622 Compounds
45%	56%	68%	76%

The two collections were filtered with FAFDrugs2 for ADMET properties, and the resulting drug-like databases were profiled using PPI-HitProfiler (D.T.1 and D.T.2 versions) to estimate the size reduction. The percentage of reduction within the table is calculated with respect to the drug-like version of the collections.

### Assessment of PPI-HitProfiler using HTS results from PubChem BioAssay

We then evaluated the performance of our PPI-HitProfiler on the HTS results of 10 different PPIs taken from the PubChem BioAssay server: BFL-1/Bid (AID432), CBFb/CBFa (AID1434), EphA4/ephrin-A (AID689), Xiap/Bir1-2 (AID1018), MCL-1/NOXA (AID1417) CD11b-CD18/Fibrinogen (AID1499), Hsp90/TPR2A (AID595), BRCT/Phosphoprotein (AID875), TLR4/MyD88 (AID811), Multiplex Bcl-2 family/Bim (AID1330). The results show a robust behavior of our tool with an average of 81% and 70% of correctly predicted PPI inhibitors and 42% and 52% of the inactive compounds removed from the initial collections when using PPI-HitProfiler-D.T.1 and –D.T.2, respectively. One can see that the D.T.2 version of PPI-HitProfiler which has a higher specificity and is therefore more appropriate for larger chemical collection shows a robust behavior for the 3 PubChem BioAssays having more than 50,000 compounds (AID1434, AID1018, and AID1499) by predicting correctly from 70 to 84% of the true PPI inhibitors while steadily removing more than half of the inactive compounds. On the other hand, for the screening assays where the total number of compounds screened is significantly below 50,000 i.e AID689, AID1417, AID811, AID1330 and for the p53/MDM2 CDithem screening (see below), one can see that the sensitivity is on average about 87% even though the average specificity on these results (38.2%) is slightly below the global average (42%). Interestingly, we noticed that true PPI inhibitors being correctly selected were flagged by the two descriptors RDF070m and UI in a 20:80 ratio highlighting the importance of multiple bonds and especially aromatic bonds in the specificity of PPI inhibitors. This further illustrates that PPI inhibitors must have a specific molecular shape, or that they tend to have a higher number of multiple bonds to compensate.

**Figure 7:**
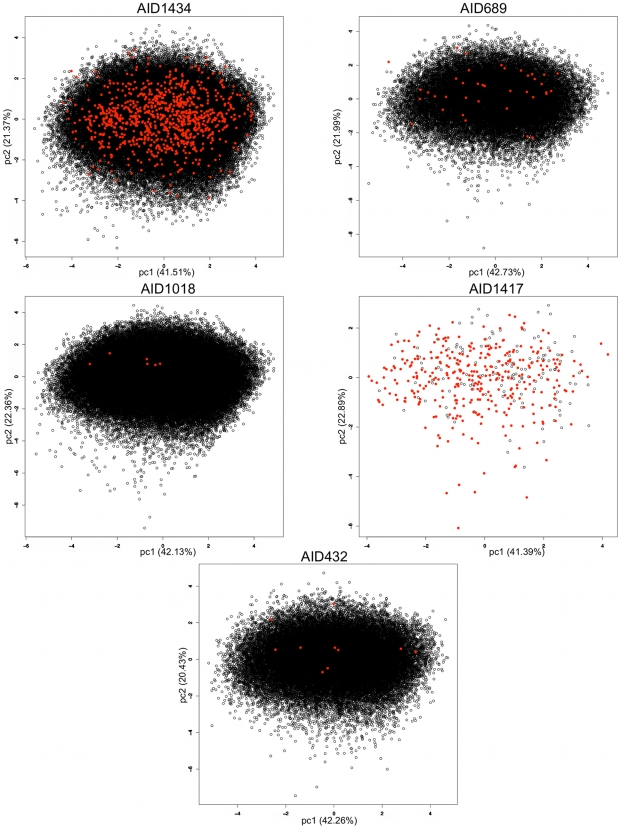
Principal Component Analysis (PCA) on the various screening results (AID1434, AID689, AID1018, AID1417, AID432). The calculations were run using 7 physico-chemical, molecular weight, octanol/water partition coefficient, topological polar surface area, number of Hydrogen bond donors and acceptors, the number of rotatable bonds and the number of rigid bonds. PPI inhibitors are represented as red disks, and non-PPI inhibitors are represented as black circles.

**Figure 8:**
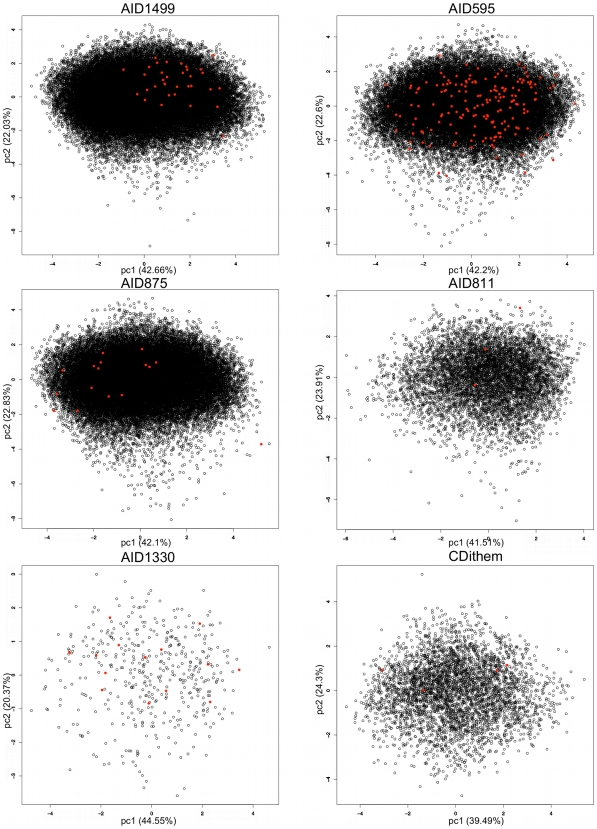
Principal Component Analysis (PCA) on the various screening results (AID1499, AID595, AID875, AID811, AID1330, CDithem). The calculations were run using 7 physico-chemical, molecular weight, octanol/water partition coefficient, topological polar surface area, number of Hydrogen bond donors and acceptors, the number of rotatable bonds and the number of rigid bonds. PPI inhibitors are represented as red disks, and non-PPI inhibitors are represented as black circles.

Similarly to what was done for the learning and validation data set, the physico-chemical PCA that was carried out on each of the AID data sets using the same 7 descriptors (Figure 7 and 8), shows that the physico-chemical subspaces spanned by the first two principal components (which account for about 60% of the variance of the global physico-chemical space) are equivalent for PPI and non-PPI inhibitors. This also means, mainly for the AIDs having a large number of active compounds (AID1434, AID689, AID1417, and AID595) for which it is easier to evaluate the level of physico-chemical space overlap (Figure 7 and 8), that classical descriptors (e.g higher MW and higher hydrophobicity, higher rigidity) may be not be always sufficient to distinguish them from inactive compounds. Rather, another way to embrace their key properties without counteracting known facts may be the further consideration of their molecular shape and aromaticity.

Finally, we also evaluated the coverage of protein space corresponding to the validation data set and the various screening results (AIDs). All combined, the validation of our tool spanned over 15 different PPIs, 5 for the validation data set and 10 for the different AID screening results. These correspond to 13 different SCOP fold classes. As it can be seen on Table 3 those classes include various types of folding including: mainly helix-based folding; mainly-beta sheet-based folding; mix-folding (helix+beta strand); and loop-binding groove systems.

**Table 3:** PPI-HitProfiler evaluation on HTS results.

Experiments	Scop fold	Nb of inatives TN + FP	Total Nb Hits TP + FN	PPI-HitProfiler	TN	TP	Sp (%)	Se (%)	EF
AID432 BFL-1/Bid	Toxins' membrane translocation domains. Multi-helical domains	46 466	10	D.T 1	20045	8	43	80	1.41
AID432 BFL-1/Bid	Toxins' membrane translocation domains. Multi-helical domains	46 466	10	D.T 2	25073	7	54	70	1.52
AID1434 CBFb/CBFa	Core binding factor beta. Barrel; capped at both ends by alpha-helices	117 533	894	D.T 1	48620	722	41	81	1.39
AID1434 CBFb/CBFa	Core binding factor beta. Barrel; capped at both ends by alpha-helices	117 533	894	D.T 2	61889	621	53	70	1.48
AID689 EphA4/ephrin-A	N/A Loop-binding groove	37 114	38	D.T 1	14684	33	40	87	1.44
AID689 EphA4/ephrin-A	N/A Loop-binding groove	37 114	38	D.T 2	18481	27	50	71	1.42
AID1018 Xiap/Bir1-2	Inhibitor of apoptosis (IAP) repeat. Metal(zinc)-bound alpha+beta fold	112 346	6	D.T 1	47084	5	42	84	1.43
AID1018 Xiap/Bir1-2	Inhibitor of apoptosis (IAP) repeat. Metal(zinc)-bound alpha+beta fold	112 346	6	D.T 2	58187	5	52	84	1.73
AID1417 MCL-1/NOXA	Toxins' membrane translocation domains. Multi-helical domains	134	347	D.T 1	58	296	43	85	5.40
AID1417 MCL-1/NOXA	Toxins' membrane translocation domains. Multi-helical domains	134	347	D.T 2	71	243	53	70	5.35
AID1499 CD11b-CD18/Fibrinogen	vWA-like. Mixed beta-sheet of 6 strands	58 790	34	D.T 1	23924	28	41	82	1.39
AID1499 CD11b-CD18/Fibrinogen	vWA-like. Mixed beta-sheet of 6 strands	58 790	34	D.T 2	29694	28	51	82	1.66
AID595 Hsp90/TPR2A	alpha-alpha superhelix. Right-handed superhelix	46 519	174	D.T 1	19309	124	42	71	1.22
AID595 Hsp90/TPR2A	alpha-alpha superhelix. Right-handed superhelix	46 519	174	D.T 2	23613	112	51	64	1.31
AID875 BRCT/Phosphoprotein	BRCT domain. Parallel. beta-sheet of 4 strands	48 183	17	D.T 1	20259	15	42	88	1.52
AID875 BRCT/Phosphoprotein	BRCT domain. Parallel. beta-sheet of 4 strands	48 183	17	D.T 2	24709	11	51	65	1.33
AID811 TLR4/MyD88	Flavodoxin-like. parallel beta-sheet of 5 strand	7 116	3	D.T 1	3502	3	49	100	1.97
AID811 TLR4/MyD88	Flavodoxin-like. parallel beta-sheet of 5 strand	7 116	3	D.T 2	4295	3	60	100	2.52
AID1330 Multiplex Bcl-2 family/Bim	Toxins' membrane translocation domains. Multi-helical domains	461	14	D.T 1	125	13	27	92	1.31
AID1330 Multiplex Bcl-2 family/Bim	Toxins' membrane translocation domains. Multi-helical domains	461	14	D.T 2	181	11	39	79	1.33
CDithem Screening p53/MDM2	SWIB/MDM2 domain. 4 helices; capped by two small 3-stranded beta-sheets	4 705	4	D.T 1	1508	3	32	75	1.10
CDithem Screening p53/MDM2	SWIB/MDM2 domain. 4 helices; capped by two small 3-stranded beta-sheets	4 705	4	D.T 2	2003	3	43	75	1.31
All HTS cumulated				D.T 1	199118	1 250	42	81	1.39
All HTS cumulated				D.T 2	248196	1 071	52	70	1.45

Results of the application of PPI-HitProfiler on topologically diverse PubChem BioAssay results and on the CDithem screening of the p53/MDM2 interaction. All data sets were previously filtered with FAF-Drugs2 using the same parameters as for the learning data set. The total number of inactive compounds (TN + FP). active compounds (TP + FN). remaining inactives (TN). and remaining actives (TP). are used to calculate the sensitivity and specificity of PPI-HitProfiler on each data set.

TP: number of PPI inhibitors correctly classified.

FP: number of non-PPI inhibitors classified as PPI-inhibitors.

TN: number of non-PPI inhibitors correctly classified.

FN: number of PPI inhibitors classified as non-PPI inhibitors.

Sensitivity = TP/(TP + FN).

Specificity = TN/(TN + FP).

### Assessment of PPI-HitProfiler through the in vitro screening of p53/MDM2

**Figure 9:**
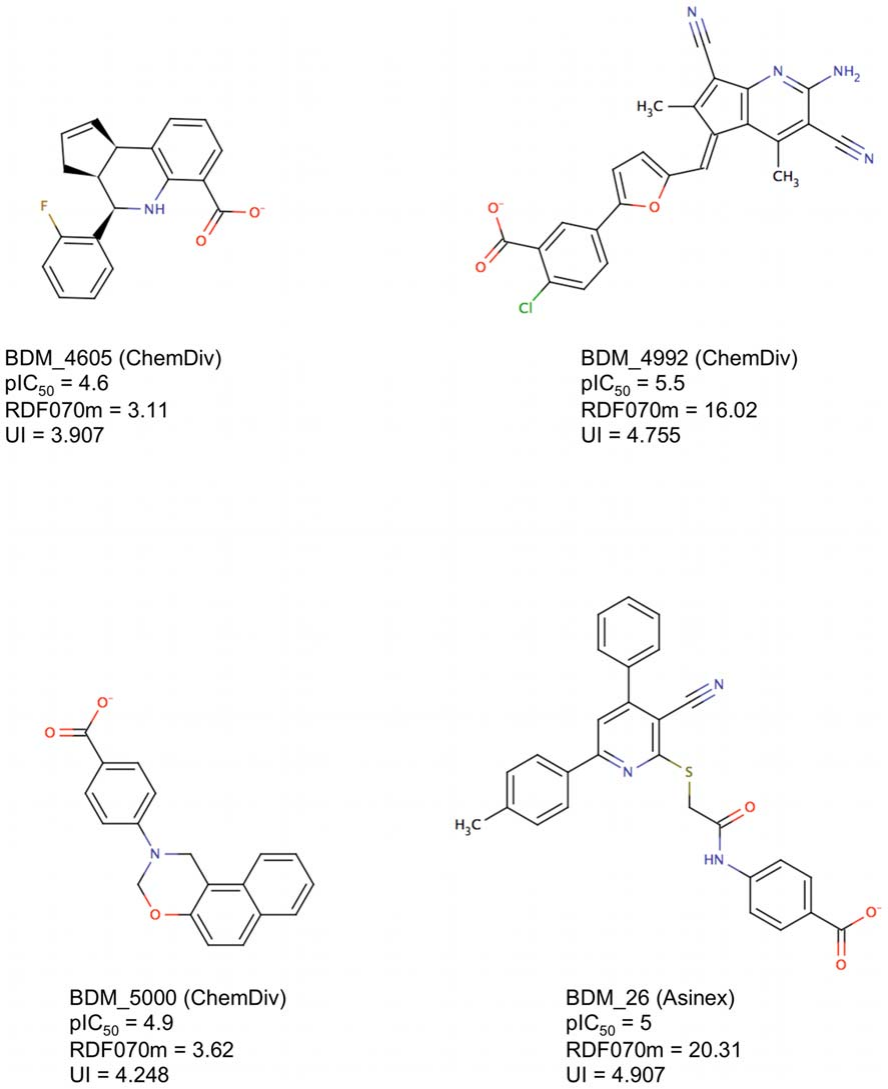
2D sketch of the 4 new inhibitors of the p53/MDM2 interaction identified by our CDithem fluorescence polarization assay along with their potency (pIC_50_) and their RDF070m and UI values.

**Figure 10:**
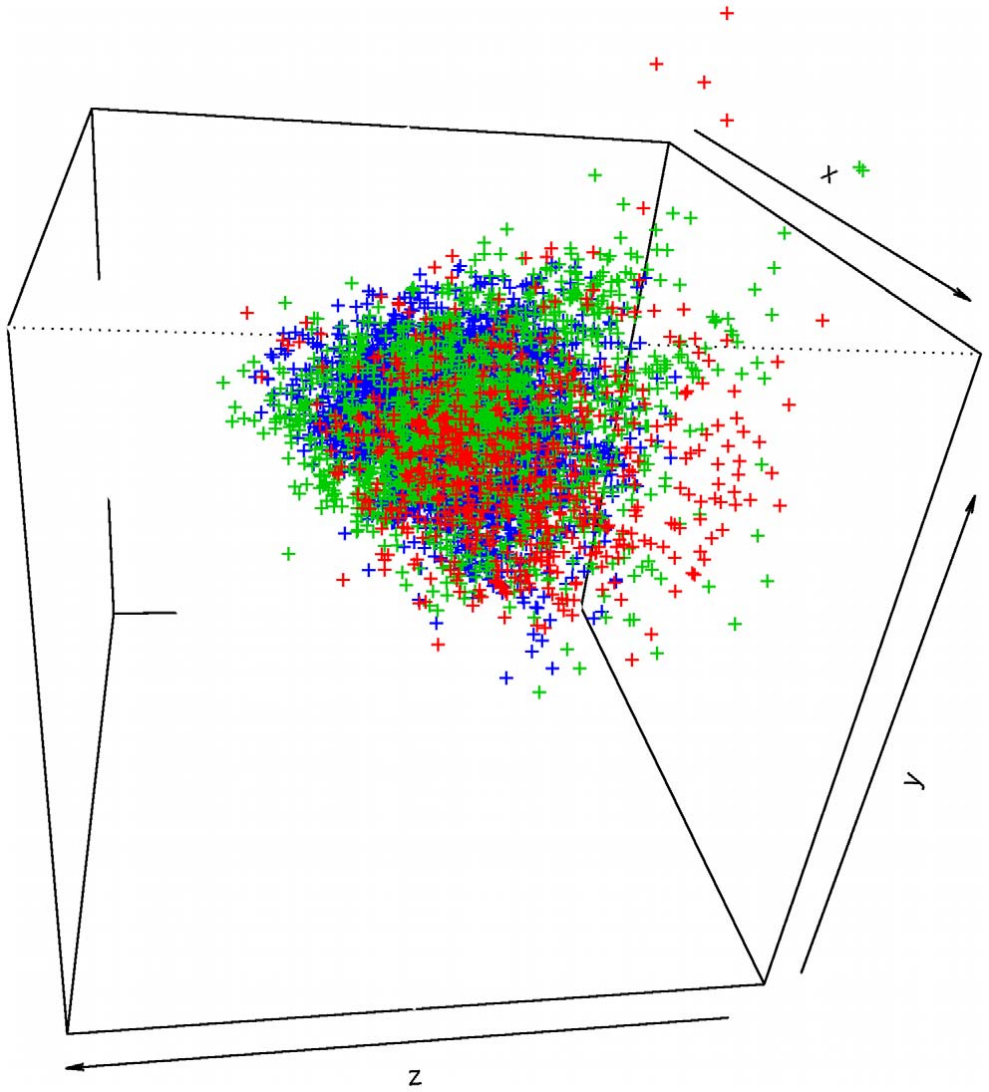
Applicability domain of PPI-HitProfiler. The application domain of PPI-HitProfiler has been evaluated using a Principal Component Analysis (PCA) on the 623 molecules of the learning data (red) set and the 357 initial E-Dragon descriptors that were used to construct the decision trees. The graph represents the 3 first axis of the PCA (40% of the variance) which have been used to calculate the coordinates of the 1,645 molecules of the ChemDiv subset (green) and the 3,060 molecules of the Asinex Subset (blue). A good overlap between the three subsets (Red, Green, and Blue) can be observed which indicates that the molecules from the Asinex and ChemDiv subsets stand within the applicability domain of PPI-HitProfiler and that the focused library resulting from the two subsets is meaningful.

We then challenged our PPI-HitProfiler through the in vitro screening of the p53/MDM2 complex. The p53 tumor suppressor is vital in cell cycle regulation DNA repair, and apoptosis[37]–[38]. Its implication has been observed in all human cancers either with mutations or through a pure inhibition due to an overexpression of its native partner, murine double minute 2 oncoprotein (MDM2). This PPI has therefore been the subject of numerous experimental screening studies yielding to the development of several synthetic PPI inhibitors [39]–[43]. Using a fluorescence polarization assay within our CDithem platform to monitor the p53/MDM2 interaction, we screened a total of 4,705 drug-like compounds filtered from Asinex (3,400 cpmds) (www.asinex.com) and ChemDiv (2,400 cpmds) (www.chemdiv.com) subsets using FAF-Drugs2. The experimental screening led to the identification of 4 new inhibitors of the p53/MDM2 interaction with pIC_50_ ranging from 4.6 to 5.5 (Figure 9). Interestingly, 3 out of those 4 new inhibitors (Se = 75%) passed our two filters (PPI-HitProfiler-D.T.1 and PPI-HitProfiler-D.T.2). Compound BDM_4605 (pIC_50_ = 4.6) was missed by both our filters because it has a low RDF070m value (3.11) far from the threshold (set at 13.31) at the top of the trees, and a low Ui value as well (3.907) when a minimal of 3.95 is required to pass at least the threshold of D.T.1. Interestingly, this represents only 14 multiple bonds (double and aromatic) when 15 are required for D.T.1. Among the three correctly detected compounds all passed by the most stringent threshold for Ui (4.13), which represents the presence of at least 17 multiple bonds. Compound BDM_26 also passed the RDF070m threshold with a value of 20.31. In this case again, one can see the star-like structure of the compound, which contributes to most of the high value of RDF070m in a similar manner to the well known structure of the Nutlin compounds (e.g compound 2 in Figure S1) that are also active on p53/MDM2. Conversely, from the 4,705 compounds tested, PPI-HitProfiler-D.T.1 managed to remove 32% of the inactive compounds (Sp = 32%) on this PPI system whereas PPI-HitProfiler-D.T.2 removed 43% (Sp = 43%) of the initial screened collection (Table 3). These results showed that used prior to experimental screening PPI-HitProfiler-D.T.2 would manage to identify 75% of the true PPI inhibitors on p53/MDM2 while nearly dividing in half the initial compound collection. As for the learning data set, the results of a physico-chemical PCA carried out on these screening results using the 7 descriptors cited above showed a fair overlap of the physico-chemical subspaces of the two subpopulations (actives and inactives). We then used the p53/MDM2 data set to assess the applicability domain of PPI-HitProfiler. We ran another principal component analysis (PCA) on the 623 molecules of the learning data set and the 357 E-Dragon descriptors that remained for the construction and optimization of the decision trees. The 3 first axis of the PCA were used to plot the molecules of the two subsets screened (Asinex + ChemDiv) in the flurorescence polarization assay (Figure 10). Even though the 3 first axis of the PCA represent only 40% of the global variance, these results tend to show that the screened collection stood within the domain of applicability of PPI-HitProfiler.

### Conclusion

In summary, we suggest that it may be possible to determine a global PPI inhibitor profile having appropriate ADMET properties using machine-learning techniques. Descriptor-based decision trees managed to positively discriminate PPI inhibitors combining only two molecular descriptors, RDF070m and Ui, which respectively describe specific ramified molecular shape and the presence of 15–17 multiple bonds in the compound. The development of a new computer package named PPI-HitProfiler allows the design of focused libraries enriched in PPI inhibitors starting from any drug-like compound collection. Its applications on two commercial compound collections, and its assessment on the experimental screening results of 11 different PPI systems shows a robust behavior in identifying true PPI inhibitors, from 70 to 81%, and its capacity to discard putative non-PPI inhibitors, from 42 to 52%, depending on the version of PPI-HitProfiler used. Although, removing 52% of inactive compounds from a chemical collection might seem rather low when designing focused chemical libraries, it has to be kept in mind that PPIs are a large and very diverse family. Futhermore, lead discovery in the pharmaceutical environment is at an industrial scale in which it is typical to screen 1–5 million compounds in few weeks using HTS. Yet the financial cost of an HTS campaign of 1 million compounds is anywhere between $500 000 to $1000 000[13]. This means in this case a cost saving from $260 000 to $2 600 000 pet target. Clearly, some potentially interesting compounds could be lost after any type of filtering but the next blockbuster can also be missed by any kind of experimental high-throughput methods. Moreover, at present no one can foresee to what extent one can reduce the initial size of screening collections using a global and target-independent PPI inhibitor profiler like the one presented herein. This really depends on the quality of the initial collection as suggests the difference in specificity between PubChem BioAssay databases, and the ChemBridge and MayBridge collections.

One avenue to circumvent this problem should be to design PPI-specific profiler that would take into account topology and types of interactions, e.g α-helix bound to a groove (p53/MDM2), or inter-protein beta-sheet (Xiap-BIR3/Smac), etc. This way, more specificity could be brought to the statistical models. One can imagine to design focused libraries by applying successive filters from the most global, like PPI-HitProfiler, to the more specific that could represent only a precise type of protein-protein interaction.

At this stage of development and present knowledge, we strongly believe that “target-independent” PPI inhibitor profiler can be successfully applied prior to in silico or in vitro screening experiments not only for drug discovery projects to avoid a full-scale screening but also for chemical biology projects. Because it is known that target selection is a major bottleneck in today's drug discovery endeavors and that targets are nowadays less validated than in the nineties [44], time and cost-effective in silico technologies could here assist achieving systematic success in spite of the present global economic downturn.

## Methods

### Data set preparation: learning and validation data sets

145 PPI inhibitors identified by both in vitro and in vivo experiments were taken from the literature and ADMET filtered with our program FAF-Drugs2[36] using very soft parameters for both physico-chemical properties and presence of toxic/reactive groups.

(100<MW<900; 0<HBD<8; 0<HBA = 12; -5<XLogP<6; 0<nROT<20; 0<TPSA<160; +one allowed Lipinski's rule violation). The remaining 81 PPI inhibitors were clustered with the program LigandInfo[45] using a hierarchical normal ascending classification with a diversity criterion of 0.8. From this classification one representative molecule from each cluster was taken such as having ultimately 66 structurally diverse PPI inhibitors (Figure S1). These compounds were used as the positive learning data set. A similar protocol was applied to define a negative learning data set. To do so, the 4,857 molecules from the “small molecules” subset of the DrugBank database were used. The small subset of the DrugBank contained for example 5% of compounds with a MW higher than 900. Moreover, not all the drugs from this subset have an orally bioavailable profile. All those molecules were therefore filtered using the same ADMET parameters. For historical reasons the small subset of the Drugbank contains very few PPI inhibitors. There are only 7 compounds from the whole “small subset” of the Drugbank that have a Tanimoto index above 0.8 with one of the 66 PPI inhibitors.

The ADME/tox filtering step selected 942 molecules that were clustered as above leading to a diversity set of 557 drug-like molecules. Ultimately, the learning data set contained 66 true PPI inhibitors, 557 non-PPI inhibitors and 623 molecules in total. An independent validation set was constructed to assess the robustness of the model. It contained 26 different PPI inhibitors (Figure S2) and 2,000 molecules from the ChemBridge diversity set filtered as above. More specifically, only two compounds from the 26 PPI inhibitors of the validation dataset had a Tanimoto indice with one of the 66 (learning dataset) PPI inhibitors comprised between 0.8 and 0.9. Very few of them came from the initial pool of 145 PPI inhibitors (3 of them). Two compounds had a Tanimoto index between 0.8 and 1.0 with of the 145 PPI inhibitors but the vast majority were some different extra compounds.

### Data set preparation: remaining data sets

The ADMET parameters used above for the filtering of the learning and validating data sets have also been used on the MayBridge and ChemBridge screening collections, as well as on the collections that were experimentally screened in the 10 PubChem BioAssays and in our fluorescence polarization assay on the p53/MDM2 interaction.

### Definition of enrichment, sensitivity and specificity

TP: number of PPI inhibitors correctly classified

FP: number of non-PPI inhibitors uncorrectly classified as PPI-inhibitors

TN: number of non-PPI inhibitors correctly classified

FN: number of PPI inhibitors uncorrectly classified as non-PPI inhibitors

EF: Enrichment factor
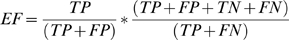


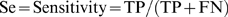


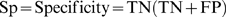



### Statistical analysis: molecular descriptor calculation and preprocessing

Descriptors were calculated by the program E-DRAGON, a web-server based version of DRAGON[28] (version 5.4) containing 1,666 descriptors.

The protocol described herein was used to eliminate non-relevant descriptors on the learning data set. Descriptors whose variance was zero (discard 108 descriptors), gathered descriptors according to correlation coefficient above 0.9 (discard 936 descriptors), descriptors whose Student T-test p-value was above 0.2 between the positive and negative learning data sets (discard 265 descriptors), such that 357 descriptors were initially retained to perform our computations.

### Learning methods

#### Support vector machines

Support vector machines belong to the class of machine learning algorithms that has recently become prominent in both computational biology and chemistry. This method implicitly embeds the data of interest in a high-dimensional feature space where classification or regression can be more easily performed with linear rules than in the original descriptor space. In SVM, a hyperplane maximizing its distance to the nearest observations (in the new space) is chosen. The optimization of parameters was processed by 10-fold cross validation (10-FCV) and factorial design. Three well-established and diverse kernels were tested: gaussian, sigmoid and polynomial. The best combination of parameters was chosen by monitoring enrichment, sensitivity and specificity from 10-FCV results (Table 4).

**Table 4:** SVM optimized parameters.

Kernel	C	Kernel Scale	Kernel offset	Kernel degree
Gaussian	10	10^−3^		
Sigmoid	10^3^	10^−4^	10^−3^	
Polynomial	10	10^−3^	1	2

#### Decision trees

Decision trees were constructed by analyzing a set of training samples for which the class labels were known. At each node, they recursively binary partition the data according to a threshold applied on one descriptor value. If trained on high-quality data, decision trees can make very accurate predictions. In this study, the decision tree was optimized with a cross validation protocol and manually edited. Instead of the classical indexes usually used for evaluating the quality of decision trees (such as entropy or Gini index), the trees were optimized such as providing the best global enrichment, which in this specific case provides a more suitable evaluation. The decision tree was built as follow. At each node, the descriptor whose best threshold value led to the best enrichment was chosen to become the local node. The construction was stopped when less than five observations were found in a leaf. Twenty trees were constructed by 20-fold cross validation. The choice of the final trees was motivated by only keeping nodes using the same descriptor for most of the trees. The final corresponding threshold was assigned to the modal value.

### p53/MDM2 interaction: Fluorescence Polarization Assay (FPA)

We chose a 9-mer peptide from p53, a fragment known to be sufficient to assess the p53/MDM2 interaction. The 9-mer p53 sequence-derived 5-carboxyfluorescein-labeled peptide (5FAM-RFMDYWEGL, Parks et *al.*, 2005) was synthesized by AnaSpec (San Jose, CA, USA).

Full length MDM2 was subcloned into the expression plasmid pET28a (Novagen, Darmstadt, Germany) using standard methods. Following protein expression in *Escherichia coli* BL21 (DE3) (Invitrogen, Carlsbad, CA, USA), bacterial cells were harvested by centrifugation followed by resuspension in 50 mM Tris-HCl (pH 8.0), 200 mM NaCl, 5 mM Imidazole, 0.1% triton X-100, protease inhibitor mixture EDTA-free (Roche Applied Science, Rotkreuz, Switzerland) at 4°C, and lysed by sonication. After centrifugation at 13000 rpm 10 min at 4°C, soluble His-tagged proteins were purified using Ni-NTA agarose beads according to the manufacturer procedures (Qiagen, Valencia, CA).

Fractions containing MDM2 proteins were pooled, dialysed into 20 mM Tris-HCl (pH 8.0), 100 mM KCl, 1 mM DTT, 0.2 mM EDTA, 0.05% triton X-100, 20% glycerol, frozen in liquid nitrogen, and kept at -80°C for further experiments.

FP assays were performed in black low-binding surface 96-well plates (Corning, NY), in a total volume of 75 µL PBS, containing 10 nM of the 5FAM-labeled peptide, 18 nM of purified MDM2, 30 µM of compound to be tested, and 3% DMSO. MDM2 was allowed to incubate with the compounds 10 min prior to adding the 5FAM-labeled peptide. After 5 minutes, FP measurements were performed on a Victor 3V plate reader (Wallac, Turku, Finland) using a 485 nm excitation filter, a 535 nm emission filter, and a 0.2 s per well reading time.

## Supporting Information

Figure S1 Chemical structures of the 66 selected PPI inhibitors used as the positive learning data set.
(1.86 MB TIF)

Figure S2 Description of the protein space coverage of the 66 PPI inhibitors of the learning data set in term SCOP fold classes. The validation data set covers 27 different PPI and 21 pairs of SCOP fold classes.
(0.04 MB PDF)

Figure S3 Chemical structures of the 26 selected PPI inhibitors used as the positive validation data set.
(0.21 MB TIF)

Figure S4 Description of the protein space coverage of the 26 PPI inhibitors of the learning data set in term SCOP fold classes. The validation data set covers 5 different PPI and 5 pairs of SCOP fold classes.
(0.02 MB PDF)
