# Supplementary figures and images for: Designing Focused Chemical Libraries Enriched in Protein-Protein Interaction Inhibitors using Machine-Learning Methods
Source: PLoS Comput Biol. 2010 Mar 5;6(3):e1000695. doi: 10.1371/journal.pcbi.1000695 (PMC2832677; doi:10.1371/journal.pcbi.1000695)

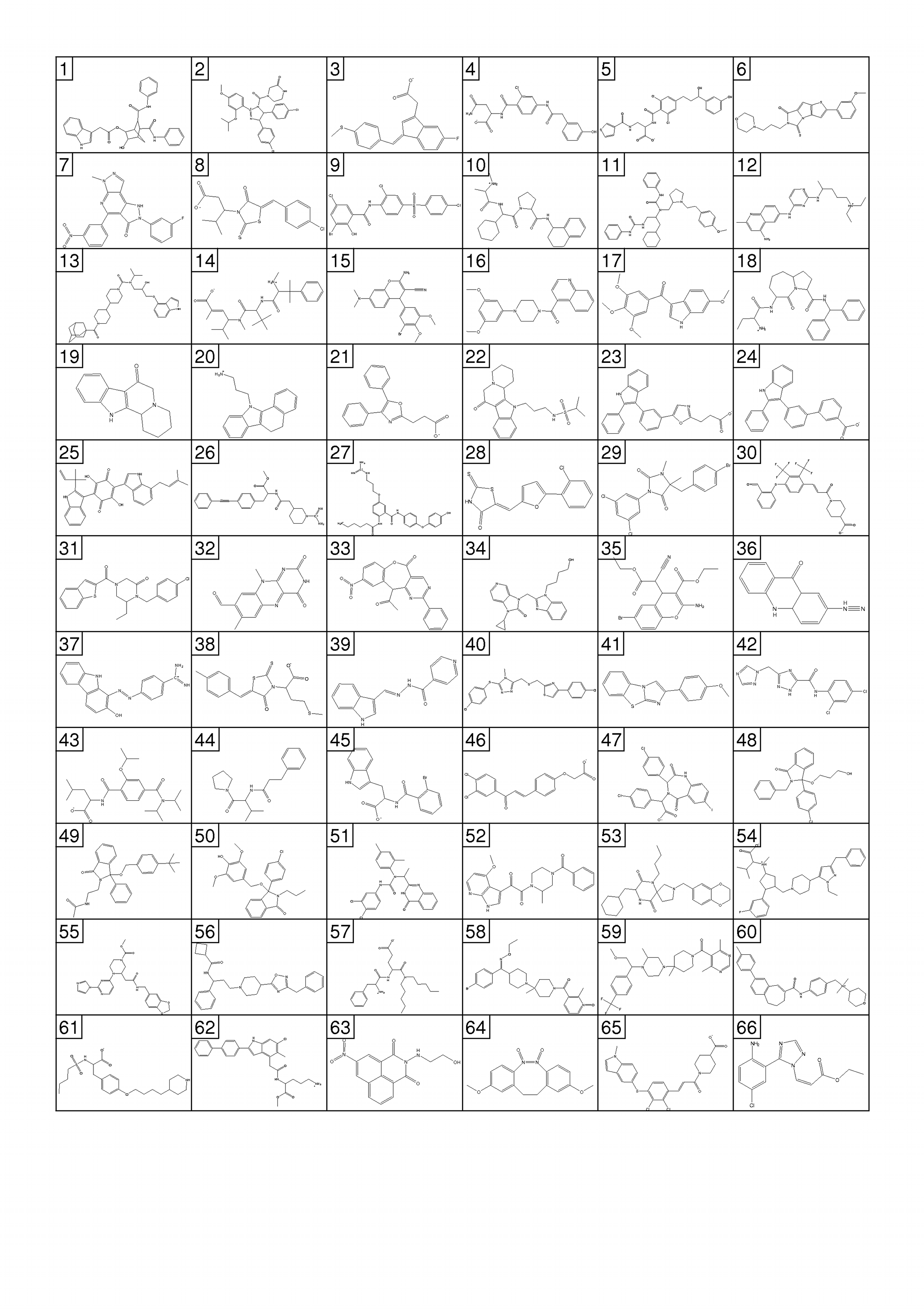

Supplement: Figure S1 — Chemical structures of the 66 selected PPI inhibitors used as the positive learning data set. (1.86 MB TIF) [file pcbi.1000695.s001.tif]

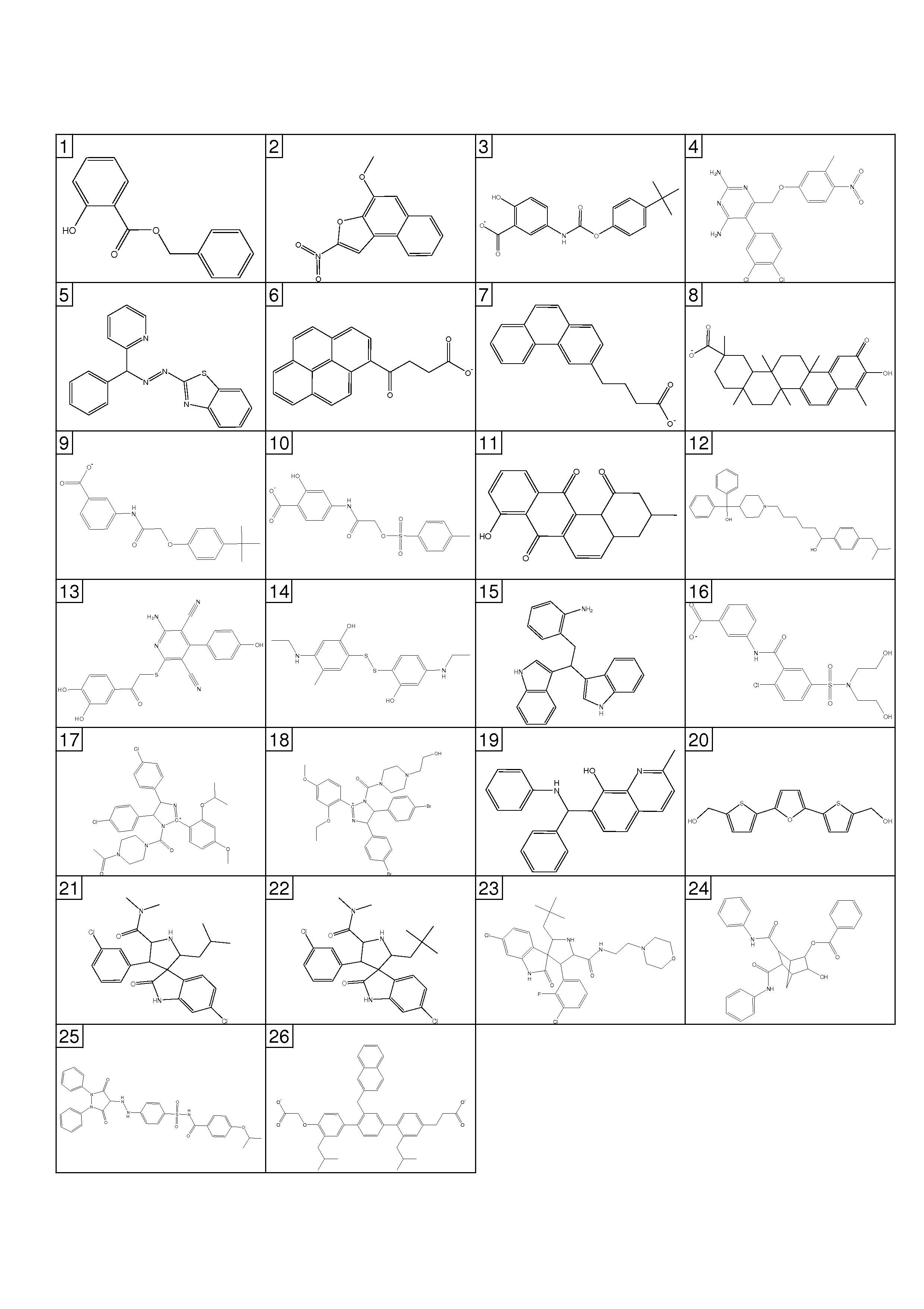

Supplement: Figure S3 — Chemical structures of the 26 selected PPI inhibitors used as the positive validation data set. (0.21 MB TIF) [file pcbi.1000695.s003.tif]
