# Supplementary material for: Designing Focused Chemical Libraries Enriched in Protein-Protein Interaction Inhibitors using Machine-Learning Methods
Source: PLoS Comput Biol. 2010 Mar 5;6(3):e1000695. doi: 10.1371/journal.pcbi.1000695 (PMC2832677; doi:10.1371/journal.pcbi.1000695)
Supplement: Figure S2 — Description of the protein space coverage of the 66 PPI inhibitors of the learning data set in term SCOP fold classes. The validation data set covers 27 different PPI and 21 pairs of SCOP fold classes. (0.04 MB PDF) [file pcbi.1000695.s002.pdf]

Ligand	PPI	SCOP FOLD
1	P53/MDM2	SWIB/MDM2 domain: core: 4 helices capped by two small 3-stranded beta-sheets
2	P53/MDM2	SWIB/MDM2 domain: core: 4 helices capped by two small 3-stranded beta-sheets
3	Ras/Raf	Cysteine-rich domain: dimetal(zinc)-bound alpha+beta fold
4	LFA-1/ICAM-1	Immunoglobulin-like beta-sandwich: 7 strands in 2 sheets
5	LFA-1/ICAM-1	Immunoglobulin-like beta-sandwich: 7 strands in 2 sheets
6	TNF-alpha/TNFRc1	TNF receptor-like : consists of three similar disulfide-rich domains
7	CD28 &CTLA4/B7.1	Immunoglobulin-like beta-sandwich: 7 strands in 2 sheets
8	Bcl-XL/Bak-BH3	Toxins' membrane translocation domains: Multi-helical domains
9	Bcl-XL/Bak-BH3	Toxins' membrane translocation domains: Multi-helical domains
10	Xiap/caspase9	Inhibitor of apoptosis (IAP) repeat: metal(zinc)-bound alpha+beta fold
11	Xiap/caspase3	Inhibitor of apoptosis (IAP) repeat: metal(zinc)-bound alpha+beta fold
12	Rac/Trio,Tiam1	Ras GEF: multihelical
13	Sur-2/ESX	N/A: alpha-helix
14	Tubulin polymerization	Tubulin nucleotide-binding domain-like: parallel beta-sheet of 6 strands
15	Tubulin polymerization	Tubulin nucleotide-binding domain-like: parallel beta-sheet of 6 strands
16	Tubulin polymerization	Tubulin nucleotide-binding domain-like: parallel beta-sheet of 6 strands
17	Tubulin polymerization	Tubulin nucleotide-binding domain-like: parallel beta-sheet of 6 strands
18	Xiap/caspase9	Inhibitor of apoptosis (IAP) repeat: metal(zinc)-bound alpha+beta fold
19	ZipA/FtsZ	TBP-like : beta-alpha-beta(4)-alpha
20	ZipA/FtsZ	TBP-like : beta-alpha-beta(4)-alpha
21	ZipA/FtsZ	TBP-like : beta-alpha-beta(4)-alpha
22	ZipA/FtsZ	TBP-like : beta-alpha-beta(4)-alpha
23	ZipA/FtsZ	TBP-like : beta-alpha-beta(4)-alpha
24	ZipA/FtsZ	TBP-like : beta-alpha-beta(4)-alpha
25	Insulin/IRTK	Protein Kinase-like
26	IL-2/IL-2R	4-helical cytokines: 4 helices
27	N-type calcium channel-multimer	N/A
28	TNF-alpha/TNFRc1	TNF receptor-like : consists of three similar disulfide-rich domains
29	LFA-1/ICAM-1	Immunoglobulin-like beta-sandwich: 7 strands in 2 sheets
30	LFA-1/ICAM-1	Immunoglobulin-like beta-sandwich: 7 strands in 2 sheets
31	LEF-1/beta-catenin	High mobility group protein: 3 helices
32	NGF-dimer	Cystine-knot cytokines : disulfide-rich fold; common core is all-beta
33	CD28 & CTLA4/B7.1	Immunoglobulin-like beta-sandwich: 7 strands in 2 sheets
34	RSV fusion protein	Stalk segment of viral fusion proteins core: trimeric coiled coil
35	Bcl-2	Toxins' membrane translocation domains: Multi-helical domains
36	Bcl-2	Toxins' membrane translocation domains: Multi-helical domains
37	Bcl-2	Toxins' membrane translocation domains: Multi-helical domains
38	Bcl-2	Toxins' membrane translocation domains: Multi-helical domains
39	CD4/MHCII	Immunoglobulin-like beta-sandwich:7 strands in 2 sheets
40	CD4/MHCII	Immunoglobulin-like beta-sandwich:7 strands in 2 sheets
41	CD4/MHCII	Immunoglobulin-like beta-sandwich:7 strands in 2 sheets
42	CD4/MHCII	Immunoglobulin-like beta-sandwich:7 strands in 2 sheets
43	Bcl-XL/bak	Toxins' membrane translocation domains: Multi-helical domains
44	IL-1-betaRI/MyD88	Immunoglobulin-like beta-sandwich: 7 strands in 2 sheets

45	LFA-1/ICAM-1	Immunoglobulin-like beta-sandwich: 7 strands in 2 sheets
46	P53/MDM2	SWIB/MDM2 domain: core: 4 helices capped by two small 3-stranded beta-sheets
47	P53/MDM2	SWIB/MDM2 domain: core: 4 helices capped by two small 3-stranded beta-sheets
48	P53/MDM2	SWIB/MDM2 domain: core: 4 helices capped by two small 3-stranded beta-sheets
49	P53/MDM2	SWIB/MDM2 domain: core: 4 helices capped by two small 3-stranded beta-sheets
50	P53/MDM2	SWIB/MDM2 domain: core: 4 helices capped by two small 3-stranded beta-sheets
51	N/A	N/A
52	Gp120/CD4	gp120 core
53	CCR5	N/A: Multi-helical domains
54	N/A	N/A
55	iNOS dimer	Nitric oxide (NO) synthase oxygenase domain: unusual fold
56	CCR5	N/A: Multi-helical domains
57	pp60c-src SH2	SH2-like :antiparallel beta-sheet of 5 strands is flanked by two helices
58	CCR5	N/A: Multi-helical domains
59	CCR5	N/A: Multi-helical domains
60	CCR5	N/A: Multi-helical domains
61	Fibrinogen/GPIIb-IIIa	Fibrinogen C-terminal domain-like:Mainly antiparallel beta sheets
62	EPO/EPOR	Immunoglobulin-like beta-sandwich:7 strands in 2 sheets
63	NGF/p75	Cystine-knot cytokines : disulfide-rich fold; common core is all-beta
64	Bcl-2/Bak-BH3	Toxins' membrane translocation domains: Multi-helical domains
65	LFA-1/ICAM-1	Immunoglobulin-like beta-sandwich: 7 strands in 2 sheets
66	CRM1/NES	alpha-alpha superhelix:alpha/alpha; right-handed superhelix
