# Supplementary material for: Designing Focused Chemical Libraries Enriched in Protein-Protein Interaction Inhibitors using Machine-Learning Methods
Source: PLoS Comput Biol. 2010 Mar 5;6(3):e1000695. doi: 10.1371/journal.pcbi.1000695 (PMC2832677; doi:10.1371/journal.pcbi.1000695)
Supplement: Figure S4 — Description of the protein space coverage of the 26 PPI inhibitors of the learning data set in term SCOP fold classes. The validation data set covers 5 different PPI and 5 pairs of SCOP fold classes. (0.02 MB PDF) [file pcbi.1000695.s004.pdf]

Ligand	PPI	SCOP Fold
1	CD81-LEL(HCV)-E2	Tetraspanin - 5 helices: irregular disulfide-linked array
2	Nef-SH3/Hck	Regulatory factor Nef - alpha(2)-beta(4)-alpha; 3 layers: alpha/beta/alpha
3	Nef-SH3/Hck	Regulatory factor Nef - alpha(2)-beta(4)-alpha; 3 layers: alpha/beta/alpha
4	Nef-SH3/Hck	Regulatory factor Nef - alpha(2)-beta(4)-alpha; 3 layers: alpha/beta/alpha
5	Nef-SH3/Hck	Regulatory factor Nef - alpha(2)-beta(4)-alpha; 3 layers: alpha/beta/alpha
6	Nef-SH3/Hck	Regulatory factor Nef - alpha(2)-beta(4)-alpha; 3 layers: alpha/beta/alpha
7	Nef-SH3/Hck	Regulatory factor Nef - alpha(2)-beta(4)-alpha; 3 layers: alpha/beta/alpha
8	Hsp90/cdc37	ATPase domain of HSP90 chaperone/DNA topoisomerase II/histidine kinase - 8-stranded mixed beta-sheet; 2 layers: alpha/beta
9	Nef-SH3/Hck	Regulatory factor Nef - alpha and beta protein
10	STAT3/STAT3 dimer	STAT-like - 4 long helices; bundle, left-handed twist (coiled coil); right-handed superhelix
11	STAT3/STAT3 dimer	STAT-like - 4 long helices; bundle, left-handed twist (coiled coil); right-handed superhelix
12	CD81-LEL(HCV)-E2	Tetraspanin - 5 helices: irregular disulfide-linked array
13	p53/MDM2	SWIB/MDM2 domain: core: 4 helices capped by two small 3-stranded beta-sheets
14	p53/MDM2	SWIB/MDM2 domain: core: 4 helices capped by two small 3-stranded beta-sheets
15	p53/MDM2	SWIB/MDM2 domain: core: 4 helices capped by two small 3-stranded beta-sheets
16	p53/MDM2	SWIB/MDM2 domain: core: 4 helices capped by two small 3-stranded beta-sheets
17	p53/MDM2	SWIB/MDM2 domain: core: 4 helices capped by two small 3-stranded beta-sheets
18	p53/MDM2	SWIB/MDM2 domain: core: 4 helices capped by two small 3-stranded beta-sheets
19	p53/MDM2	SWIB/MDM2 domain: core: 4 helices capped by two small 3-stranded beta-sheets
20	p53/MDM2	SWIB/MDM2 domain: core: 4 helices capped by two small 3-stranded beta-sheets
21	p53/MDM2	SWIB/MDM2 domain: core: 4 helices capped by two small 3-stranded beta-sheets
22	p53/MDM2	SWIB/MDM2 domain: core: 4 helices capped by two small 3-stranded beta-sheets
23	p53/MDM2	SWIB/MDM2 domain: core: 4 helices capped by two small 3-stranded beta-sheets
24	p53/MDM2	SWIB/MDM2 domain: core: 4 helices capped by two small 3-stranded beta-sheets
25	p53/MDM2	SWIB/MDM2 domain: core: 4 helices capped by two small 3-stranded beta-sheets
26	p53/MDM2	SWIB/MDM2 domain: core: 4 helices capped by two small 3-stranded beta-sheets
